# Exploratory study of associations between monetary reward anticipation brain responses and mu-opioid signalling in alcohol dependence, gambling disorder and healthy controls

**DOI:** 10.1016/j.ynirp.2024.100211

**Published:** 2024-06-27

**Authors:** Samuel Turton, Louise M. Paterson, James FM. Myers, Inge Mick, Chen-Chia Lan, John McGonigle, Henrietta Bowden-Jones, Luke Clark, David J. Nutt, Anne R. Lingford-Hughes

**Affiliations:** a Division of Psychiatry, Imperial College London, London, UK; b Department of Neuroimaging, Institute of Psychiatry, Psychology & Neuroscience, Kings College London, London UK; c Department for Psychiatry and Psychotherapy, Psychiatric University Clinic of Charité at St. Hedwig Hospital, Berlin, Germany; d Department of Psychiatry, Taichung Veterans General Hospital, Taichung Taiwan; e Perspectum Ltd, Oxford, UK; f National Problem Gambling Clinic, London UK; g Division of Psychology and Language Sciences, UCL, London UK; h Department of Psychiatry, Cambridge University, Cambridge, UK; i Centre for Gambling Research at UBC, Department of Psychology, University of British Columbia, Vancouver Canada; j Djavad Mowafaghian Centre for Brain Health, University of British Columbia, Vancouver Canada

## Abstract

Alcohol dependence (AD) and gambling disorder (GD) are common addiction disorders with significant physical and mental health consequences. AD and GD are associated with dysregulated responses to reward which could be due to a common mechanism of dysregulated endogenous opioid signalling. We explored associations between reward anticipation responses, using the Monetary Incentive Delay (MID) functional magnetic resonance imaging (fMRI) task, and mu-opioid receptor (MOR) availability and endogenous opioid release capacity using [^11^C]carfentanil positron emission tomography (PET), in 13 AD, 15 GD and 14 heathy control (HC) participants. We also examined differences in MID task reward anticipation responses between AD, GD and HC participants. These were secondary exploratory analysis of data collected to examine differences in MOR PET in addiction. We did not find significant differences in MID win > neutral anticipation BOLD responses compared between participant groups in a priori ROIs (ventral striatum, putamen, caudate) or whole brain analyses. We found no significant correlations between MID win > neutral anticipation BOLD responses and [^11^C]carfentanil PET measures, except for limited negative correlations between putamen MOR availability and MID win > neutral anticipation BOLD response in AD participants. Previous research has suggested a limited role of endogenous opioid signalling on MID task reward anticipation responses in AD and HCs as these responses are not modulated by opioid receptor blockade and this may explain our lack of significant correlations in HC and AD or GD participants. Our results, particularly the lack of differences in MID win > neutral anticipation BOLD responses across participants groups, may be limited due to only including AD or GD participants who are abstinent or in active treatment.

## 1 Introduction

Alcohol dependence (AD) and gambling disorder (GD) are common addiction disorders associated with high levels of comorbid mental and physical health problems. (Castillo-Carniglia et al., 2019; Firth et al., 2019). However, available treatments are limited and have poor long-term effectiveness (Potenza et al., 2019; Witkiewitz et al., 2019) leaving a substantial unmet treatment need.

AD and GD have common features including impaired control of alcohol use or gambling, increased precedence of the addiction over other usual activities and interests and escalation of alcohol use or gambling despite negative consequences (World Health Organization, 2019). They also both have features of dysregulated reward sensitivity, striatal reward processing and striatal dopaminergic signalling (Balodis and Potenza, 2015; Boileau et al., 2014; Luijten et al., 2017; Martinez et al., 2005). These features may represent common mechanistic targets for treating AD and GD. For example opioid receptor antagonists, such as naltrexone, are treatments in both AD (Murphy et al., 2022) and GD (Kraus et al., 2020).

Endogenous opioid signalling plays a key role in reward processing, particularly via mu-opioid receptors (MORs) (Berridge and Kringelbach, 2015). We have shown blunted endogenous opioid release capacity in both AD and GD (Mick et al., 2016; Turton et al., 2020) using [^11^C]carfentanil, a MOR selective positron emission tomography (PET) ligand (Vardanyan and Hruby, 2014). These changes in MOR signalling may contribute to the dysregulated striatal dopaminergic signalling shown in AD (Martinez et al., 2005) and GD (Boileau et al., 2014) and be a potential target for opioid receptor antagonist treatment. Better understanding the relationship between MORs and striatal dopaminergic function in addiction may test this hypothesis and provide an insight into the mechanism of opioid receptor antagonist treatment.

The Monetary Incentive Delay (MID) functional magnetic resonance imaging (fMRI) task is widely used to probe mesocorticolimbic signalling in addictive disorders (Balodis and Potenza, 2015; Luijten et al., 2017; Qiu and Wang, 2021; Zeng et al., 2023a, Zeng et al., 2023b; Zeng et al., 2023a, Zeng et al., 2023b). fMRI BOLD signal change during MID reward anticipation (i.e. win > neutral anticipation contrast) has been shown to be correlated with PET measures of striatal dopaminergic signalling, although the evidence for this is mixed (Schott et al., 2008; Urban et al., 2012; Weiland et al., 2014). Therefore, the dysregulated reward anticipation observed in some MID task studies in AD and GD (Clark et al., 2019; Luijten et al., 2017; Zeng et al., 2023a, Zeng et al., 2023b) may be a marker of the dysregulated endogenous dopamine release shown in these addictions (Boileau et al., 2014; Martinez et al., 2005; Narendran et al., 2014). The present study sought to examine the opioidergic basis of this dysregulation via the associations with [^11^C]carfentanil PET measures. Recently published data have shown no significant associations between MOR availability and MID task reward anticipation BOLD in healthy controls or antipsychotic treated individuals with schizophrenia (Shatalina et al., 2023), however they did not explore associations between MID reward anticipation and endogenous opioid release capacity. Our current study examined these associations in AD and GD rather than schizophrenia.

To investigate associations between opioidergic and mesocorticolimbic reward anticipation activity, we combined [^11^C]carfentanil PET and MID fMRI task data collected in the same participants with AD or GD and healthy controls (HC). [^11^C]carfentanil is a MOR selective PET ligand which has been used to investigate MOR availability in a range of addictions including alcohol (Bencherif et al., 2004; Heinz et al., 2005; Turton et al., 2020) and cocaine dependence (Ghitza et al., 2010; Gorelick et al., 2008) and gambling disorder (Mick et al., 2016). [^11^C]carfentanil PET can be used to measure endogenous opioid release (Manninen et al., 2017; Scott et al., 2007), including following an oral dexamphetamine challenge (Colasanti et al., 2012; Mick et al., 2014). We therefore selected [^11^C]carfentanil PET to examine MOR availability and endogenous opioid release capacity in both alcohol dependence and gambling disorder and the data used in this paper was originally collected for these studies (Mick et al., 2016; Turton et al., 2020). In addition to [^11^C]carfentanil PET scans our participants also completed an fMRI scanning session which included the MID task. This provided a unique dataset of both MOR PET and fMRI measures in the same participants with two different types of addiction and allowed secondary exploratory analyses examining associations between opioidergic signalling and reward processing in addiction.

Our primary focus was the combined [^11^C]carfentanil PET and MID fMRI data to examine associations between dysregulated reward anticipation responses, MOR availability, and dexamphetamine-induced endogenous opioid release capacity in addiction. We hypothesised that lower MOR availability, reflecting a lower sensitivity of endogenous opioid signalling, would be associated with blunted win > neutral anticipation BOLD contrast in AD and GD. We also hypothesised that lower endogenous opioid release capacity would be associated with lower win > neutral anticipation BOLD contrast, reflecting a broader reward deficit state.

We also examined the MID fMRI data separately where we tested the hypothesis that monetary reward anticipation responses would be lower in AD and higher in GD participants, reflecting the lower and higher striatal dopamine release in these addictions respectively (Boileau et al., 2014; Martinez et al., 2005; Narendran et al., 2014).

## 2 Methods

### 2.1 Study design

Male participants with AD (n = 13), GD (n = 15) and HCs (n = 14) were recruited as part of the addiction neuroimaging program at the Centre for Neuropsychopharmacology, Imperial College London. AD was diagnosed using DSM-5 and GD using DSM-IV criteria (*see Supplementary Methods*). Only male subjects were recruited due to the evidence of sex-related differences in MOR availability (Kantonen et al., 2020) and potential effects of different phases of the menstrual cycle on MOR availability (Smith et al., 1998). All participants completed an fMRI protocol developed for a previous study (McGonigle et al., 2017; Paterson et al., 2015) that included an MID task. Participants completed two [^11^C]carfentanil PET scans, one before and the other 3 h following a 0.5 mg/kg oral dose of dexamphetamine (Mick et al., 2016; Turton et al., 2020). The fMRI protocol and [^11^C]carfentanil PET scans were completed on separate visits, typically two weeks apart. This was to eliminate any impact of endogenous opioid release associated with completing the fMRI tasks, including the MID task, on our [^11^C]carfentanil PET measures. Participants were required to provide a negative alcohol breathalyser and urine drug screen on all study visits.

### 2.2 Participants

Our participants have been previously characterised (Mick et al., 2014, 2016; Turton et al., 2020), but will be outlined here. All were male. AD participants were recruited from NHS and associated clinical services in North West London, UK, and were abstinent from alcohol for at least 4 weeks. GD participants were recruited from the NHS National Problem Gambling Clinic, Central North West London NHS Foundation Trust London, UK either prior to or during an 8-week course of CBT. HCs were recruited by advertisement and had no previous mental health history or substance or gambling addictions, with the exception of nicotine. ADs and GDs with any other addiction history, except nicotine and alcohol or gambling respectively, were excluded. Previous anxiety and depression in AD and GD participants were allowed due to a high prevalence of these disorders in these groups, but participants with current anxiety and/or depression or any past or present history of other mental health diagnoses were excluded.

### 2.3 PET methods

[^11^C]carfentanil PET methods have been previously detailed (Colasanti et al., 2012; Mick et al., 2014, 2016; Turton et al., 2020). All participants underwent [^11^C]carfentanil PET scans at the Imanova/Invicro clinical imaging centre, Hammersmith hospital, London, on the same Siemens HiRez Biograph 6 PET/CT scanner (Siemens Healthcare, Erlangen, Germany). After positioning in the scanner, a head-fixation device (soft strap) was used to reduce head movements during the scan. A low dose CT scan was performed immediately prior to each [^11^C]carfentanil PET scan for attenuation and scatter correction. Participants received a 20 ml saline infusion over 20 s containing up to 350 MBq [^11^C]carfentanil via an antecubital vein cannula. The maximum cold mass of carfentanil was limited to 0.3 μg/kg to reduce any active pharmacological effects.

Dynamic [^11^C]carfentanil emission data were collected continuously for 90 min, consisting of 26 frames: 8 × 15 s frames, 3 × 60 s frames, 5 × 120 s frames, 5 × 300 s frames and 5 × 600 s frames. Listmode data were reconstructed by the Siemens HiRez Biograph 6 PET/CT scanner with filtered back projection (discrete inverse Fourier transform, with a 128 matrix, 2.6 zoom and 5 mm 3-dimensional transaxial Gaussian filter, full width at half maximum – FWHM). Reconstruction included scatter, attenuation, dead-time, random detections and radioactive decay corrections.

The dynamic PET image data analysis was carried with MIAKAT (www.miakat.org). Each frame was corrected for radioactive decay and then for head movement parameters by rigid-body co-registration to the 16th frame as a reference. The summed PET image was then rigid-body co-registered to the subject's volumetric T1-weighted magnetisation-prepared rapid acquisition gradient-echo sequence (MPRAGE) MRI.

The MPRAGE structural MRI was used to derive non-linear transformation parameters of the stereotaxic Clinical Imaging Centre (CIC) atlas, developed on the Montreal Neurological Institute (MNI) 152 non-linear 6th generation average brain template (Grabner et al., 2006) using SPM12 unified segmentation (Statistical Parametric Mapping - www.fil.ion.ucl.ac.uk/spm). These non-linear transformation parameters were used to non-linearly co-register the CIC atlas to individual participant's PET data space to extract time activity curve (TAC) data for each ROI. The ROI's TAC data was then used to estimate specific regional [^11^C]carfentanil binding (BP_ND_) using the simplified reference tissue model (SRTM) (Hirvonen et al., 2009; Lammertsma and Hume, 1996) with an eroded occipital lobe white matter mask for the reference tissue.

The fractional reduction in [^11^C]carfentanil BP_ND_ following dexamphetamine was calculated in each ROI as: Δ*BP*_*ND*_=(*BP*_*NDpost*_−*BP*_*NDpre*_)/*BP*_*NDpre*_. This variable was used as an index for endogenous opioid release capacity.

### 2.4 fMRI protocol

Our fMRI protocol has been previously published in detail (McGonigle et al., 2017; A. Murphy et al., 2017; Nestor et al., 2017; Paterson et al., 2015). All MRI data were collected on the same 3 T S Tim Trio scanner at the Imanova/Invicro clinical imaging centre, Hammersmith hospital, London, running syngo MR B17 software and using a Siemens 32 channel receive-only phased-array head coil.

Each session consisted of localizer scans, main magnetic field mapping, one run of a resting state task, two runs of the MID task, two runs of a go-nogo task and two runs of an evocative images task. Structural scans were carried out after the tasks and included a high resolution MPRAGE scan for anatomical registration. Participants were in the scanner for approximately 80 min. All tasks were practiced outside the scanner shortly before the start of the MRI scanning session.

fMRI imaging used multi-echo gradient echoplanar imaging with the following parameters: TR = 2000ms, TE = 13ms and 31ms, flip angle = 80°, field of view = 225 mm, image matrix = 64 × 64), in-plane resolution of 3.516 × 3.516 mm and a slice thickness of 3.000 mm. The direction of phase encoding was anterior to posterior, echo spacing was 0.52ms. The second echo (TE = 31ms) only was used for the fMRI analysis. 36 abutting oblique axial slices were collected for each volume in an ascending manner at a 30° angle to the anterior and posterior commissure line. GRAPPA with a 2 x acceleration factor was performed for parallel imaging. The first three volumes of each functional run were discarded to allow for T1 saturation effects.

### 2.5 MID task protocol

The MID task had an event-related design with fixed win/loss amounts of £0.50 in addition to a neutral cue (i.e. zero outcome). The task consisted of 36 win trials, 36 neutral trials and 12 loss trials, delivered over two task runs. It used blocks of several TRs in length (each run lasting 7 min 12 s with combined total of 14 min 24 s).

At the start of each trial participants were shown an on screen cue indicating the trial type (i.e. win, lose or neutral trial). After the cue, an ‘anticipation period’ blank black screen with variable duration (randomly 2, 3 or 4 s) was shown. The target stimulus was then shown requiring participants to respond by pressing a button box whilst the target symbol remained on the screen to ‘hit’ the target. Three possible outcomes following a successful hit were: winning £0.50 in a win trial, preventing the loss of £0.50 in a lose trial or nothing in a neutral trial. The target stimulus duration time varied throughout the task depending on the subjects performance. For each trial type the initial the target presentation time was 280ms and would either reduce by 10ms after a successful hit or increase by 10ms following a miss up to a minimum of 150ms or maximum of 300ms. The duration of the target symbol for each trial type was dependent on performance only to the same trial type (i.e. successful hits to a win trial would only affect win trial target duration time). The algorithm modifying target presentation duration times aimed for an accuracy for the win trials of 60–70% and approximate total winnings of £10 for the combined two task runs (out of a potential maximum of £18).

Participants received performance feedback (hit or miss) 0.5 s following the target symbol and their current winnings for the two trials up for 2 s. There was a variable interval (2.4, 3.4, or 4.4 s) between the end of the outcome/winnings information and the next cue.

There was an imbalance in the number of win and loss trials (36 and 12 respectively) designed into the task to prioritise the power of the win > neutral main contrast of interest in the analyses. Subsequently, due to low power the loss trial was not included as a contrast of interest. Also, the task was designed for participants to achieve approximately 60–70% accuracy, therefore resulting in too low power to explore successful vs unsuccessful outcome contrasts for each trial type.

### 2.6 fMRI analysis

All pre-processing of fMRI and MID task data were carried out using the methods described by McGonigle et al. (2017). Structural and functional MRI pre-processing included the following toolboxes: AFNI (Analysis of Functional NeuroImages – version AFNI_2011_12_21_1014), FreeSurfer (version freesurfer-x86_64- unknown-linux-gnu-stable5-20130513), ANTs (Advanced Normalization Tools – version ANTs-1.9.v4-Linux), and FSL's (FMRIB Software Library – version 5.0.6) FEAT (FMRI Expert Analysis Tool – version 6.00) run on CentOS 6.5 (version centos-release-6- 5.el6.centos.11.2.x86_64).

T1 images were corrected for intensity non-uniformity (AFNI 3dUniformize) and then brain extracted (FreeSurfer recon-all pipeline). Images were then non-linearly registered to the MNI 152 non-linear 6th generation brain template in 2 mm isotropic voxel space using ANTs antsRegistration.

Echoplanar images (EPIs) were corrected for slice timing effects (using AFNI 3dTshift) before each volume was registered (using AFNI 3dvolreg) to the volume most similar to all others (in-house code). Extracerebral tissues were removed (FSL BET) and linear registration to the T1 image used Boundary Based Registration (BBR with FSL epi_reg). EPIs were transformed into the same standard stereotaxic space as the transformed T1 (using ANTs antsApplyTransforms). Data were smoothed with a three-dimensional Gaussian kernel of full width at half maximum of 6.0 mm.

### 2.7 MID task modelling

Pre-whitening was performed (FMRIB Improved Linear Modelling - FILM) and estimates of six head movement parameters (three orthogonal directions and pitch, roll and yaw) were calculated (AFNI 3dvolreg) and along with movement outliers (scan-to-scan displacement of >3 mm) were included in each model as confounding explanatory variables. Mean task head movement data was recorded as velocity over the entire task (mm/s). The haemodynamic response function (HRF) convolution used a mean lag of 6 s and standard deviation of 3 s.

‘Anticipation’ was modelled as a block starting at cue presentation and ending at the target onset (blocks lasting approximately 3 s–5 s). The two runs of the MID task for each subject were modelled separately in FEAT and then averaged across both runs to produce the contrast used in ROI and ‘higher-level’ whole-brain analyses. The contrast of interest used for ROI and higher-level analyses is ‘win anticipation’ > ‘neutral anticipation’. FSL FLAME (FMRIB's Local Analysis of Mixed Effects) was used for whole-brain FLAME analyses. Win > neutral anticipation BOLD contrast percent change values (%BOLD) were extracted as a mean for each ROI using FSL FEATquery.

MID task behavioural data (accuracy and response time, as well as task-related head movement parameters) were checked to ensure all participants responded to the MID task appropriately. One HC was excluded from further analyses due to poor task performance (0% neutral trial accuracy).

### 2.8 Group-level data analyses

We used three a priori ROIs (Fig. S1) to test for differences in win > neutral anticipation BOLD contrast between the groups, and the associations between MOR availability/endogenous opioid release capacity and win > neutral anticipation BOLD contrast within each participant group; the nucleus accumbens (NAcc), caudate and putamen.

These regions have both high MOR and dopamine receptor availability (Kantonen et al., 2020; Malén et al., 2022) and play an important role in the endogenous opioid mediation of reward responses (Berridge and Kringelbach, 2015; Le Merrer et al., 2009). They form a key part in MID task reward anticipation BOLD contrast (Wilson et al., 2018) and this is correlated with dopamine release in the NAcc and putamen (Schott et al., 2008; Urban et al., 2012).

Dysregulated MID task anticipation BOLD has been demonstrated in these regions in a arrange of addictions (Balodis and Potenza, 2015; Clark et al., 2019; Zeng et al., 2023a, Zeng et al., 2023b), which may reflect evidence of dysregulated dopamine receptor availability and dopamine release (Ashok et al., 2017; Boileau et al., 2014; Heinz et al., 2004; Martinez et al., 2005) also seen in these regions.

Given the above, these regions are useful to investigate the link between opioidergic signalling and MID reward anticipation BOLD responses, potentially as a correlate of dopaminergic signalling, in addiction. More broadly, correlations between MOR availability and task related BOLD signalling have also been previously demonstrated in these regions (Karjalainen et al., 2017, 2019).

All PET and fMRI ROIs were defined using the CIC neuroanatomical atlas (Tziortzi et al., 2011). The CIC atlas was designed for PET image analysis and ROIs have binary categorisation with no overlap between adjacent ROIs (i.e. a single voxel is defined as either 100% or 0% within a single specific ROI).

### 2.9 Statistics

One-way ANOVAs were used to examine demographic variables across the three participant groups, with pairwise analyses using independent-samples t-tests. Chi-squared tests examined differences in the frequencies of smokers between the three groups. For ROI analyses, two-tailed t-tests were carried out to compare the %BOLD values in a-priori ROIs between participant groups. Pearson's correlation coefficients were used to examine associations between MOR availability (BP_ND_), endogenous opioid release capacity (ΔBP_ND_) and win > neutral anticipation %BOLD signal change across the three ROIs in each subject group separately. These analyses were carried out using IBM SPSS (version 28 for Mac).

Exploratory whole-brain two-tailed *t*-test FLAME analyses examined differences in win > neutral anticipation BOLD between the participant groups. Whole-brain analyses examined correlations between [^11^C]carfentanil BP_ND_ and win > neutral anticipation BOLD by using ROI [^11^C]carfentanil BP_ND_ or ΔBP_ND_ values as an explanatory variable of interest in the GLM model. A cluster defining threshold (CDT) of Z > 3.1 was used for all whole-brain FLAME analyses to reduce the risk of false positive results (Eklund et al., 2016).

## 3 Results

### 3.1 Demographics and task behaviour

There was higher mean age in AD participants compared with GD participants, but neither group differed significantly from the HC. A somewhat larger proportion of AD participants were current smokers compared with GD participants and HCs, but this was not significant (Table 1).

**Table 1:** Demographic data (mean ± SD) compared between healthy controls, alcohol dependent and gambling disorder participants.

Variable	Healthy control	Alcohol dependent	Gambling disorder	p-value
Number of participants	13	13	15	N/A
Age (yrs)	39.7 (±11.8)	46.6 (±7.3)^b^	34.3 (±7.3)†	**0.003**
BMI	25.1 (±4.6)	26.7 (±3.7)	26.8 (±5.0)	0.535
Alcohol abstinence (days)	11.5 (±21.6)^a^	605 (±867)^a^,^b^	8.9 (±7.9)†	**0.003**
Gambling abstinence (days) (GD only)	N/A	N/A	43.1 (±42.6)	N/A
Current smokers	3 (23%)	7 (54%)	4 (27%)	0.190

p-values are results for one-way ANOVAs, with the exception of Current Smokers % where the p-value is a chi-squared test.

Post-hoc tests (Mann Whitney *U* test for alcohol abstinence and independent sample *t*-test for all other variables).

N/A – not applicable.

** HC vs. GD p < 0.05.

a HC vs. AD p < 0.05.

b AD vs. GD p < 0.05.

MID task behavioural data showed no statistically significant differences in performance between the groups (see Table S1). AD participants had significantly higher head movement during the task compared with GD participants, but the AD participants did not differ from HCs, and no participants met a threshold for excess head movement to require exclusion (Supplementary Methods).

### 3.2 fMRI win > neutral anticipation BOLD responses

**Fig. 1:**
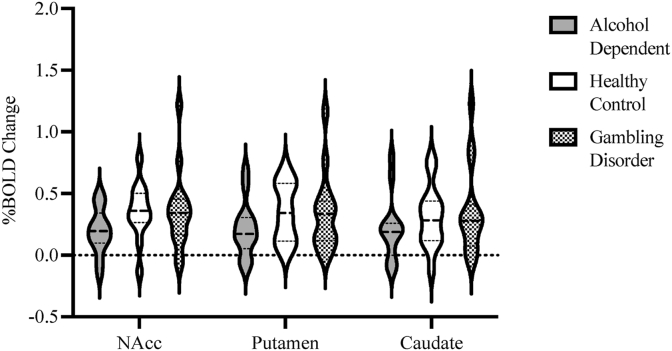
Violin plot (with median) of percent BOLD signal change for win > neutral anticipation contrast in healthy controls, alcohol dependent and gambling disorder participants across 3 ROIs.

Differences in win > neutral anticipation BOLD contrast between the three participant groups were examined across the three ROIs (Fig. 1). Two-tailed t-tests comparing %BOLD change between groups were non-significant (all p > 0.05). Whole-brain FLAME two-tailed independent sample t-tests comparing win > neutral anticipation BOLD contrast between groups (AD > HC, AD<HC, GD>HC, HC > GD, AD > GD and GD > AD) did not show any significant clusters at a CDT of Z > 3.1.

There were no significant correlations between MID win > neutral anticipation %BOLD change and duration of abstinence from alcohol or gambling in either AD or GD participants respectively (Pearson's correlation coefficient, all uncorrected p > 0.05).

Associations between [^11^C]carfentanil BP_ND_, ΔBP_ND_ and MID win > neutral anticipation.

Associations between [^11^C]carfentanil BP_ND_, ΔBP_ND_ and MID win > neutral anticipation BOLD contrast (%BOLD signal change) were examined using Pearson's correlations in the three ROIs (Table S2A) in each subject group separately.

There were no significant correlations between [^11^C]carfentanil BP_ND_ and %BOLD signal change in HCs or in GD participants. In AD participants, there were negative correlations between putamen [^11^C]carfentanil BP_ND_ and %BOLD signal change in NAcc, putamen and caudate (R = −0.613, p = 0.026, R = −0.688, p = 0.009 and R = −0.657, p = 0.015 respectively) (Fig. S2, Table S2A). Significant correlation analyses were repeated with bootstrapping (1000 samples) (Table S2B).

There were no significant correlations between [^11^C]carfentanil ΔBP_ND_ (i.e. endogenous opioid release in response to the amphetamine challenge) and %BOLD signal change in any participant group (Table S2C).

**Fig. 2:**
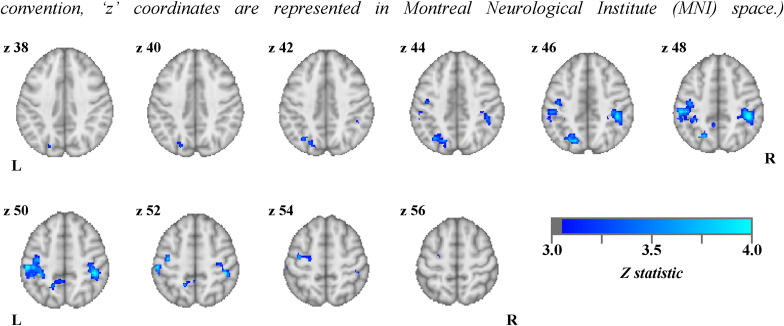
Significant (Z > 3.1, p < 0.05) **negative** correlations between putamen [^11^C]carfentanil BP_ND_ and MID win > neutral anticipation BOLD contrast in **alcohol dependent participants** (oriented by neurological convention, ‘z’ coordinates are represented in Montreal Neurological Institute (MNI) space.).

The trend associations between MID win > neutral anticipation BOLD and putamen [^11^C]carfentanil BP_ND_ in AD participants (Table S2A) were explored using a FLAME whole brain analysis. Putamen regional BP_ND_ values were entered as an explanatory variable of interest in a GLM model (AD participants only). There was a significant negative correlation between putamen [^11^C]carfentanil BP_ND_ and MID win > neutral anticipation BOLD contrast in 3 cortical clusters in AD participants (Z > 3.1, p < 0.05), including the right and left post-central and supramarginal gyri, left lateral occipital cortex and precuneus (Fig. 2, Table S3).

## 4 Discussion

We hypothesised that our exploratory analyses would show MID win > neutral anticipation BOLD contrast was lower in AD, and higher in GD, compared with the healthy comparison group, reflecting the dysregulated striatal dopaminergic signalling in these addictions. We did not find any significant between-group differences in this contrast, either in our ROI or whole-brain analyses. As such, these data fail to corroborate some other evidence of dysregulated MID reward-related activity in AD and GD, though there is well-recognized inconsistency in the significance and direction of this dysregulation in the published literature (Balodis and Potenza, 2015; Clark et al., 2019; Luijten et al., 2017; Zeng et al., 2023a, Zeng et al., 2023b). Similar to our findings, one prior study that directly compared AD and GD participants showed no significant differences in win > neutral anticipation BOLD contrast (Romanczuk-Seiferth et al., 2015). Differences in findings across the literature are likely due to variation in durations of abstinence, inclusion of participants with other substance dependence co-morbidity, or differences in the value of the win (or loss) amounts (Clark et al., 2019; Luijten et al., 2017). A large voxel-wise meta-analysis of monetary reward showing evidence of blunted anticipation BOLD responses in alcohol dependence included a mix of studies with short- or long-term abstinence (Zeng et al., 2023a, Zeng et al., 2023b). Our participants have long abstinence durations, typically months to years, and the largest study in a similar population with established abstinence also shows no significant differences in monetary reward anticipation BOLD responses in AD compared with controls (Musial et al., 2023). Our sample size was smaller than most other studies’ samples which may affect the power of our analyses as discussed below.

We hypothesised that lower MOR availability would be associated with lower MID win > neutral anticipation BOLD contrast, and that lower endogenous opioid release capacity (i.e. ΔBP_ND_ calculated from pre- and post-dexamphetamine [^11^C]carfentanil scans) would be associated with lower MID win > neutral anticipation BOLD contrast. We observed correlations between higher putamen MOR availability and lower striatal win > neutral anticipation BOLD responses within the AD group. We did not correct for multiple comparisons due to the exploratory nature of our analyses and note these effects would not survive such a correction.

Whole-brain exploratory analysis in AD participants also showed correlations between higher putamen MOR availability and lower win > neutral anticipation BOLD contrast in supramarginal, pre- and post-central gyrii, superior parietal lobule, precuneus and lateral occipital cortex. These cortical regions have a role in motor, sensory, visual and spatial processing (Johns, 2014) and play a role in the MID task win > neutral anticipation BOLD contrast (Wilson et al., 2018), likely reflecting their role in the processing of the visual cues and preparation of motor responses during the anticipation period. There is evidence of projections from these cortical regions to the putamen in humans (Cacciola et al., 2017), which also has a key role in motor function (Cummings, 1993). The MID task has a significant motor element during the anticipation period (i.e. preparation for a rapid motor response), that may be orchestrated in the dorsal striatum.

In contrast to our hypothesised positive correlations between MOR availability and MID task BOLD contrast, negative correlations have also been shown in healthy subjects using vicarious pain and emotional arousal tasks (Karjalainen et al., 2017, 2019), but across a much wider range of regions than in our results. We hypothesised that higher MOR availability would indicate greater MOR receptor density or affinity and therefore greater sensitivity to endogenous opioid signalling. However, [^11^C]carfentanil PET is an indirect measure of MOR density and higher BP_ND_ could instead result from lower endogenous opioid binding to MORs, possibly due to blunted endogenous opioid release capacity as we have previously shown in AD and GD (Mick et al., 2016; Turton et al., 2020). Subsequent lower endogenous opioid ligand concentrations would result in higher MOR availability but reflect lower sensitivity of endogenous opioid signalling.

We did not observe significant correlations between win > neutral anticipation BOLD contrast and our MOR PET measures in HCs or GD, with our only results limited to negative correlations in AD participants. We did not formally test for differential effects in AD participants compared with GD participants or HCs (i.e. interaction analyses to detect differences in the correlation coefficients). It may be that our result in AD represents the impact of a confounding variable which affects both endogenous opioid and striatal dopaminergic signalling in AD participants, for example the toxic effect of chronic heavy alcohol use, rather than the modulation of win > neutral anticipation BOLD contrast by MOR signalling in this group.

Our results, particularly in HCs, broadly replicate recently published results finding no significant associations between MID reward anticipation BOLD and MOR availability (Shatalina et al., 2023). Shatalina et al. also found no associations between MOR availability and reward anticipation BOLD in individuals with schizophrenia who had blunted reward anticipation responses. However, these individuals were prescribed antipsychotic medication which significantly complicates the interpretation of these results, particularly given the impact of dopamine receptor blockade on MID reward anticipation responses (Hawkins et al., 2021). Our AD and GD participants were not taking any psychotropic medication at the time of their participation.

Our fMRI and PET scans occurred on different days, and it is not known whether engagement in the MID task is sufficient to drive endogenous opioid signalling, either in the healthy brain or states of addiction. This would limit the role of MORs in modulating dopaminergic striatal signalling during the task. Our previous findings that naltrexone does not modulate MID win > neutral anticipation BOLD in AD participants or HCs (Nestor et al., 2017) may corroborate the hypothesis of a lack of active endogenous opioid signalling during the MID task.

Endogenous opioid signalling, except in regions responsible for cardiorespiratory regulation, is phasic (Gestreau et al., 2000). The studies that found significant associations between MOR availability and BOLD signal used tasks related to pain and emotional arousal (Karjalainen et al., 2017, 2019; Scott et al., 2007) and both pain and invoked emotional response paradigms can also be used to induce phasic endogenous opioid release (Manninen et al., 2017; Scott et al., 2007). The presence of task induced endogenous opioid signalling during these paradigms may explain their observed associations between MOR availability and BOLD signal. However, in our study a lack of MID task induced endogenous opioid signalling during the task could explain the minimal associations between win > neutral anticipation BOLD and our measures of MOR availability and endogenous opioid release capacity.

One interesting task to examine associations between striatal BOLD signalling and MOR availability in addiction would be addiction-related cue responses. Striatal BOLD responses to salient alcohol cues in AD participants are blunted by opioid receptor antagonist naltrexone (Karl et al., 2021; Schacht et al., 2017) suggesting these cues activate phasic endogenous opioid signalling. The modulation of this tasks’ striatal BOLD responses by naltrexone is a predictor of treatment outcome in AD (Bach et al., 2020, 2021) and therefore examining the associations between MOR availability and addiction cue response BOLD signal would be important to understanding of the mechanism of the clinical effect of naltrexone in addiction.

### 4.1 Limitations

The studies in which our participants were scanned were designed around the PET [^11^C]carfentanil PET measurements and were not powered for the analyses presented here. The sample sizes of studies examining group differences in MID win > neutral anticipation BOLD contrast in addiction (Chen et al., 2022; Luijten et al., 2017) would suggest at least 20 participants in each group are required. Previous HC studies examining associations between [^11^C]carfentanil PET and BOLD contrast in vicarious pain and emotional arousal tasks recruited 33 and 35 participants respectively (Karjalainen et al., 2017, 2019). Post-hoc power calculations suggest that our dataset was underpowered for both the win > neutral anticipation BOLD comparison between groups and MID BOLD and [^11^C]carfentanil BP_ND_ correlation analyses (Supplementary Section). Low power increases the risk of both type-I and type-II errors (Button et al., 2013) which further highlights the exploratory nature of these analyses and the caution required when interpreting our results.

Given the evidence of the significant role of endogenous opioid signalling in reward consummation (Berridge and Kringelbach, 2015), the ‘outcome’ phase of the MID task could be of more interest than the anticipation phase. As discussed in the methods section, due to the design of our MID task, we did not have adequate power to use the outcome phase in our analyses. Furthermore, the MID outcome phase BOLD signal may not reflect ‘reward consummation’, but instead a prediction error signal (Oldham et al., 2018), or ‘spillover’ of anticipatory phase BOLD signal (Demidenko et al., 2021).

We did not correct for multiple comparisons and most, if not all, of our a priori regional results in AD participants would not be significant following an appropriate correction. As discussed in the introduction, this is an exploratory secondary analysis of data collected to investigate MOR PET measures in addiction. However, given the unique dataset of both [^11^C]carfentanil PET and MID win anticipation responses in the same AD and GD participants, we believe there is still value in presenting these analyses to inform further research investigating the association between opioidergic and dopaminergic signalling in addiction.

Despite our experience working in this field, recruiting AD, GD and HC participants was challenging, and our groups were not perfectly matched on key demographic variables including age and smoking status. There is also a difference in the months to years duration of abstinence in AD participants compared with the days to weeks abstinence duration in GD participants. Abstinence may be an important factor in abnormal MOR availability in addiction, for example AD participants with short periods of abstinence have high MOR availability (Heinz et al., 2005) whilst those with longer durations do not (Turton et al., 2020). Although higher MOR availability has not been demonstrated in recently abstinent GD participants (Mick et al., 2016). Our GD participants were engaged with treatment services, whilst our AD participants were not, and this may yield differential results related to the effects of treatment.

There was a higher proportion of current smokers in the AD participant group. Nicotine dependence has been shown to be associated with differences in MID reward anticipation responses (Balodis and Potenza, 2015) and there is evidence of differences in MOR availability associated with smoking (Nuechterlein et al., 2016), compared with non-smokers. Our numbers were too small to explore the impact of nicotine in each of our three groups so we are unable to exclude any contribution from an unmatched number of smokers on our results.

We used different spatial data processing methods for our PET and fMRI data. This difference is likely to introduce some spatial variation when comparing between the two modalities, particularly in small volumes (i.e. voxel to voxel level). However, there are already a number of important factors affecting the spatial comparability of BOLD and PET signals prior to our pre-processing stage. This includes scanner modality dependent differences in spatial resolution (Jamadar et al., 2020; Moses, 2011), PET partial volume effects (Rousset et al., 1998) and on-scanner attenuation correction and smoothing (see Methods). These factors would significantly affect the reliability of small volume spatial equivalence between the two modalities even if identical spatial pre-processing methods were used. For these reasons we prioritised the quality of our ROI values using our established methods for each modality and used large volume ROIs for between scan analyses to minimise any volume effects of using different methods.

## 5 Conclusion

In summary we have shown exploratory evidence of no associations between MOR availability and win > neutral anticipation BOLD contrast in GD participants or HCs and only minimal associations in AD participants. Further studies with adequately powered samples are required to confirm these results. Our findings may suggest that endogenous opioid signalling has little role during anticipation of a monetary reward in the MID task and would be in keeping with our previous findings that opioid receptor blockade with naltrexone does not mediate MID win > neutral anticipation BOLD responses in AD participants or HCs (Nestor et al., 2017). Further research using an fMRI behavioural paradigm that induces phasic opioid release may allow us to examine associations between MOR availability and dopaminergic signalling in gambling disorder and alcohol dependence. Improving our understanding of such neurobiology would help the development of better or more focussed treatments in these addictions.

## Funding statement

This article presents independent research funded by the 10.13039/501100000265Medical Research Council (Grant G1002226) and 10.13039/100013216Imperial College BRC and carried out at the National Institute for Health and Care Research (10.13039/501100000272NIHR) 10.13039/100013216Imperial Clinical Research Facility (CRF). The views expressed are those of the authors and not necessarily those of the Medical Research Council, Imperial College BRC, the NHS, the NIHR or the Department of Health and Social Care.

## Ethics approval statement

This study was approved by the West London Research Ethics Committee (14/LO/1552) and the Administration of Radioactive Substances Advisory Committee, UK. The research in this article was carried out in accordance with The Code of Ethics of the World Medical Association (Declaration of Helsinki) for experiments involving humans.

## Patient consent statement

Written informed consent was obtained from all the participants.

## CRediT authorship contribution statement

**Samuel Turton:** Writing – review & editing, Writing – original draft, Project administration, Methodology, Investigation, Formal analysis, Data curation, Conceptualization. **Louise M. Paterson:** Writing – review & editing, Supervision, Methodology, Formal analysis. **James FM. Myers:** Writing – review & editing, Supervision, Methodology, Investigation, Formal analysis, Data curation, Conceptualization. **Inge Mick:** Writing – review & editing, Project administration, Investigation, Data curation. **Chen-Chia Lan:** Writing – review & editing, Methodology, Investigation. **John McGonigle:** Writing – review & editing, Methodology, Formal analysis. **Henrietta Bowden-Jones:** Writing – review & editing, Investigation. **Luke Clark:** Writing – review & editing, Investigation, Conceptualization. **David J. Nutt:** Writing – review & editing, Writing – original draft, Supervision, Resources, Investigation, Funding acquisition, Conceptualization. **Anne R. Lingford-Hughes:** Writing – review & editing, Writing – original draft, Supervision, Methodology, Investigation, Funding acquisition, Formal analysis, Data curation, Conceptualization.

## Declaration of competing interest

Prof Luke Clark is the Director of the Centre for Gambling Research at 10.13039/501100005247UBC, which is supported by funding from the Province of British Columbia and the British Columbia Lottery Corporation (BCLC), a Canadian Crown Corporation. The Province of BC government and the BCLC had no role in the design, analysis, or interpretation of the study, and impose no constraints on publishing. LC has received remuneration from the International Center for Responsible Gaming (travel; speaker honoraria; academic services), the Institut fur Glucksspiel und Gesellschaft (Germany; travel; speaker honoraria), GambleAware (UK; academic services), Gambling Research Australia (academic services), Alberta Gambling Research Institute (Canada; travel; academic services), German Foundation for Gambling Research (advisory board; travel). He has been remunerated for legal consultancy by the BCLC. He has not received any further direct or indirect payments from the gambling industry or groups substantially funded by gambling. LC receives an honorarium for his role as Co-Editor-in-Chief for International Gambling Studies from Taylor & Francis, and he has received royalties from Cambridge Cognition Ltd. relating to neurocognitive testing. Prof David Nutt is the CRO of Awakn Life Sciences, chair of DrugScience [UK] and PAREA Europe, a member of the Lundbeck Foundation Neurotorium programme and chair of its editorial board, advisor to the British National Formulary and a member of the International Centre for Science in Drug Policy. He has received grants or clinical trial payments from Wellcome Trust, MRC, COMPASS Pathways and Usona, speaking honoraria from Janssen, Lundbeck, Otsuka and Rovi, has share options in P1vital, Awakn, Psyched Wellness and Neurotherapeutics and is a director of Equasy Enterprises and GABA Labs. He has been an expert witness in a number of legal cases relating to psychotropic drugs, is editor of the Drug Science Policy and Law Journal and has edited/written 38 books, some of which have been purchased by pharmaceutical companies.Prof Anne Lingford-Hughes reports honoraria for speaking engagements and/or attendance at conferences from 10.13039/501100013327Lundbeck, 10.13039/501100013327Lundbeck Institute UK, 10.13039/100004319Pfizer and Servier; she has received research grants or support from Alcarelle and Lundbeck; she is a member of the 10.13039/501100000544British Association for Psychopharmacology and receives honoraria for speaking at educational events and led their ‘addiction’ guidelines, which received support from Archimedes Pharma, 10.13039/501100013327Lundbeck, 10.13039/100004319Pfizer and Schering.The other authors declare no conflicts of interest in relation to this paper.

## Data Availability

Unthresholded z-statistic maps for fMRI analysis from this paper are available on Neurovault.org (https://identifiers.org/neurovault.collection:14230). Other data available on reasonable request
