# Supplementary material for: Exploratory study of associations between monetary reward anticipation brain responses and mu-opioid signalling in alcohol dependence, gambling disorder and healthy controls
Source: Neuroimage Rep. 2024 Jun 27;4(3):100211. doi: 10.1016/j.ynirp.2024.100211 (PMC11427764; doi:10.1016/j.ynirp.2024.100211)
Supplement: Multimedia component 1 [file mmc1.docx]

**SUPPLEMENTARY INFORMATION**

*SUPPLEMENTARY METHODS*

*Diagnostic Criteria*

Alcohol dependence was diagnosed using DSM-5 criteria for ‘severe’ alcohol use disorder (American Psychiatric Association, 2013) which requires the presence of 6 or more of the following symptoms:

Have you?

- Had times when you ended up drinking more, or longer, than you intended?
- More than once wanted to cut down or stop drinking, or tried to, but couldn’t?
- Spent a lot of time drinking? Or being sick or getting over other aftereffects?
- Wanted a drink so badly you couldn’t think of anything else?
- Found that drinking—or being sick from drinking—often interfered with taking care of your home or family? Or caused job troubles? Or school problems?
- Continued to drink even though it was causing trouble with your family or friends?
- Given up or cut back on activities that were important or interesting to you, or gave you pleasure, in order to drink?
- More than once gotten into situations while or after drinking that increased your chances of getting hurt (such as driving, swimming, using machinery, walking in a dangerous area, or having unsafe sex)?
- Continued to drink even though it was making you feel depressed or anxious or adding to another health problem? Or after having had a memory blackout?
- Had to drink much more than you once did to get the effect you want? Or found that your usual number of drinks had much less effect than before?
- Found that when the effects of alcohol were wearing off, you had withdrawal symptoms, such as trouble sleeping, shakiness, restlessness, nausea, sweating, a racing heart, or a seizure? Or sensed things that were not there?

Gambling disorder was diagnosed using the DSM-IV criteria for ‘Pathological Gambling” (American Psychiatric Association, 2000) requiring 5 or more of the following symptoms:

- Is preoccupied with gambling (e.g., preoccupied with reliving past gambling experiences, handicapping or planning the next venture, or thinking of ways to get money with which to gamble)
- Needs to gamble with increasing amounts of money in order to achieve the desired excitement
- Has repeated unsuccessful efforts to control, cut back, or stop gambling
- Is restless or irritable when attempting to cut down or stop gambling
- Gambles as a way of escaping from problems or of relieving a dysphoric mood (e.g., feelings of helplessness, guilt, anxiety, depression)
- After losing money gambling, often returns another day to get even (“chasing” one’s losses)
- Lies to family members, therapist, or others to conceal the extent of involvement with gambling
- Has committed illegal acts such as forgery, fraud, theft, or embezzlement to finance gambling
- Has jeopardized or lost a significant relationship, job, or educational or career opportunity because of gambling
- Relies on others to provide money to relieve a desperate financial situation caused by gambling
- The gambling behaviour is not better accounted for by a Manic Episode.

*Quality control of pre-processed PET and MRI data and MID task modelling*

Non-linear transformations (both SPM12 unified segmentation and ANTs antsRegistration) and of structural MRI, EPI and PET data were visually inspected for adequate fit for further analysis if fMRI and PET data. MID task movement parameters (average movement per volume in mm/s) were plotted for participants assess for extreme movement (i.e. >10% of volumes having a scan-to-scan displacement >3mm). MID task behavioural data was checked to check appropriate participant response to the tasks. One healthy control participant was excluded due to poor task performance (0% neutral trial accuracy).

SUPPLEMENTARY RESULTS

***Figure S1*** *– Putamen, caudate and NAcc ROIs from the CIC neuroanatomical atlas and used for [^11^C]carfentanil PET and win>neutral anticipation BOLD ROI analyses (oriented by neurological convention, ‘z’ coordinates are represented in Montreal Neurological Institute (MNI) space.)*

| ***Table S1*** *– MID task behavioural data (mean ±SD) compared between healthy controls, alcohol dependent and gambling disorder participants* | | | | |
| --- | --- | --- | --- | --- |
| Variable | Healthy control | Alcohol dependent | Gambling disorder | p value |
| Total amount won | £9.54 (±1.56) | £9.69 (±2.28) | £10.63 (±1.84) | 0.266 |
| Win task accuracy | 66.0% (±4.7) | 67.5% (±6.9) | 69.8% (±5.7) | 0.237 |
| Neutral task accuracy | 54.7% (±18.1) | 63.0% (±7.6) | 63.0% (±8.6) | 0.142 |
| Lose task accuracy | 60.9% (±14.2) | 59.0% (±18.8) | 67.8% (±14.7) | 0.314 |
| Win task reaction time (ms) | 235.5 (±19.1) | 225.6 (±21.2) | 221.8 (±20.0) | 0.202 |
| Neutral task reaction time (ms) | 253.8 (±29.5) | 238.8 (±25.4) | 243.5 (±25.0) | 0.352 |
| Lose task reaction time (ms) | 232.5 (±22.8) | 227.2 (±24.7) | 222.1 (±23.8) | 0.520 |
| Mean MID task head movement (mm‎/s) | 0.086 (±0.058) | 0.108 (±0.045)*** | 0.059 (±0.016)*** | 0.016 |

*All statistical tests in this table are one-way ANOVAs*

*Post-hoc independent sample two-tailed t-test:*

** AD vs. GD p<0.05*

***Figure S2*** *– Correlations between Putamen [^11^C]carfentanil BP_ND_ and (A) NAcc, (B) putamen and (C) caudate percent BOLD signal change for win>neutral anticipation contrast in alcohol dependent participants (including Pearson’s R and p value)*

| ***Table S2A*** *– Correlation coefficients (Pearson’s R) between [^11^C]carfentanil BP_ND_ and MID win>neutral anticipation %BOLD signal change, in Healthy Controls, Alcohol Dependent and Gambling Disorder.* | | | |
| --- | --- | --- | --- |
| MID %BOLD signal change ROI | Healthy Controls | Alcohol Dependent | Gambling Disorder |
|  | NAcc BP_ND_ Pearson’s R values | | |
| NAcc | -0.169 | -0.050 | 0.104 |
| Putamen | -0.249 | -0.134 | 0.127 |
| Caudate | -0.124 | 0.072 | 0.069 |
|  | Putamen BP_ND_ Pearson’s R values | | |
| NAcc | 0.254 | -0.613* | -0.239 |
| Putamen | 0.212 | -0.688** | -0.353 |
| Caudate | 0.148 | -0.657* | -0.362 |
|  | Caudate BP_ND_ Pearson’s R values | | |
| NAcc | 0.201 | -0.304 | -0.018 |
| Putamen | 0.090 | -0.239 | -0.048 |
| Caudate | 0.144 | -0.002 | -0.024 |

p<0.05, p<0.01

| ***Table S2B*** *– Correlation coefficients (Pearson’s R) between Putamen [^11^C]carfentanil BP_ND_ and MID win>neutral anticipation %BOLD signal change in Alcohol Dependence with bootstrap (1000 samples) 95% confidence intervals* | | | |
| --- | --- | --- | --- |
| MID %BOLD signal change ROI | Pearson’s R value | Bootstrap R value 95% CI | P value |
| NAcc | -0.613 | -0.845 to -0.175 | 0.026 |
| Putamen | -0.688 | -0.932 to 0.143 | 0.009 |
| Caudate | -0.657 | -0.910 to -0.035 | 0.015 |

| ***Table S2C*** *– Correlation coefficients (Pearson’s R) between [^11^C]carfentanil ∆BP_ND_ and MID win>neutral anticipation %BOLD signal change, in Healthy Controls, Alcohol Dependent and Gambling Disorder participants.* | | | |
| --- | --- | --- | --- |
| MID %BOLD signal change ROI | Healthy Controls | Alcohol Dependent | Gambling Disorder |
|  | NAcc ∆BP_ND_ Pearson’s R values | | |
| NAcc | -0.018 | 0.213 | -0.078 |
| Putamen | 0.044 | 0.384 | -0.146 |
| Caudate | -0.184 | 0.350 | -0.029 |
|  | Putamen ∆BP_ND_ Pearson’s R values | | |
| NAcc | -0.031 | 0.059 | -0.200 |
| Putamen | -0.003 | 0.009 | -0.266 |
| Caudate | -0.003 | -0.031 | -0.314 |
|  | Caudate ∆BP_ND_ Pearson’s R values | | |
| NAcc | -0.022 | -0.098 | -0.004 |
| Putamen | 0.018 | -0.091 | -0.085 |
| Caudate | -0.048 | -0.232 | -0.155 |

| ***Table S3*** *– Negative correlations between MID win>neutral anticipation BOLD and Putamen [^11^C]carfentanil BP_ND_ in Alcohol Dependent participants* | | | | | | |  |
| --- | --- | --- | --- | --- | --- | --- | --- |
| Regions | Cluster voxels | p | Local maxima Z score | MNI coordinates of maxima (mm) | | | |
|  |  |  |  | x | y | z | |
| Cluster 1 (Left) | 402 | 0.0002 |  |  |  |  | |
| Postcentral gyrus, Supramargnal gyrus |  |  | 4.09 | -52 | -28 | 50 | |
| Precentral gyrus |  |  | 3.92 | -38 | -16 | 52 | |
| Postcentral gyrus |  |  | 3.84 | -42 | -28 | 50 | |
| Superior parietal lobule, Postcentral gyrus |  |  | 3.79 | -34 | -38 | 50 | |
| Superior parietal lobule, Postcentral gyrus |  |  | 3.73 | -38 | -38 | 50 | |
| Postcentral gyrus |  |  | 3.65 | -32 | -34 | 50 | |
| Cluster 2 (Right) | 286 | 0.0020 |  |  |  |  | |
| Supramarginal gyrus, Postcentral gyrus |  |  | 4.14 | 42 | -34 | 48 | |
| Supramarginal gyrus |  |  | 3.13 | 54 | -34 | 50 | |
| Cluster 3 (Left) | 207 | 0.0107 |  |  |  |  | |
| Lateral occipital cortex |  |  | 3.93 | -16 | -68 | 46 | |
| Lateral occipital cortex |  |  | 3.85 | -20 | -62 | 48 | |
| Lateral occipital cortex |  |  | 3.63 | -30 | -66 | 44 | |
| Precuneus |  |  | 3.62 | -14 | -52 | 52 | |
| Precuneus |  |  | 3.41 | -6 | -48 | 48 | |
| Lateral occipital cortex |  |  | 3.4 | -18 | -74 | 42 | |

Coordinates (in MNI space) and Z score maxima for cluster-based statistical contrasts (all Z>3.1, p<0.05). Local maxima are named according to the structure(s) at that position in the Harvard-Oxford Cortical Atlas.

**Post-Hoc power calculations**

Post-hoc power calculations using mean ROI win>neutral anticipation BOLD values suggest sample sizes ranging from approx. n=20 to n=50 in each group to detect differences between AD and HC or GD. Sample sizes required to detect differences between HC and GD were all n>1000 in each group (power=0.8 and alpha=0.05). Post-hoc power calculation using our significant correlations between MID win anticipation and putamen BP_ND_ [^11^C]carfentanil in AD participants suggests a range between n=14 (putamen %BOLD Pearson’s r=0.688) to n=18 (NAcc %BOLD Pearson’s r=0.613) subjects in each group (power=0.8 and alpha=0.05).

**SUPPLEMENTARY REFERENCES**

American Psychiatric Association. (2000). *Diagnostic and statistical manual of mental disorders (4th ed., text rev.).*

American Psychiatric Association. (2013). *Diagnostic and Statistical Manual of Mental Disorders, 5th Edition (DSM-5)*.
